# Sustained Delivery of Chondrogenic Molecules Using Sugar Glass Nanoparticle-Integrated Fibrous Scaffolds for Cartilage Tissue Engineering

**DOI:** 10.1007/s40883-025-00472-3

**Published:** 2025-08-27

**Authors:** To Wang, Yiwei Dong, Marcus T. Cicerone, Wan-Ju Li

**Affiliations:** 1 Department of Orthopedics and Rehabilitation, University of Wisconsin-Madison, 1111 Highland Ave. WIMR 5051, Madison, WI 53705 USA; 2 Department of Biomedical Engineering, University of Wisconsin-Madison, 1550 Engineering Drive, Madison, WI 53706 USA; 3 Department of Chemistry and Biochemistry, Georgia Institute of Technology, 950 Atlantic Drive, Atlanta, GA 30332 USA

**Keywords:** Nanoparticle, Drug delivery, Electrospinning, Tissue engineering, Mesenchymal stem cell, Chondrogenesis

## Abstract

**Purpose:**

Biomaterial scaffolds capable of controlled release of bioactive molecules hold significant potential in tissue engineering, offering a promising avenue to enhance tissue regeneration. They provide localized and sustained delivery of biological cues to direct stem cell differentiation while creating a three-dimensional microenvironment that supports cell adhesion and growth.

**Methods:**

In this study, we utilized reverse micelle sugar glass nanoparticles (SGnPs), previously developed by our team, to encapsulate the chondrogenic growth factor TGFB1. This approach aimed to preserve the bioactivity of these molecules before their release. The TGFB1-SGnPs were directly incorporated into electrospun fibrous scaffolds, engineered specifically to ensure the sustained release of the growth factor during the culture of human bone marrow-derived mesenchymal stem/stromal cells (BMSCs).

**Results:**

TGFB1 was released in a sustained manner over 39 days from TGFB1-SGnP-incorporated fibrous scaffolds, made from poly (ε-caprolactone), poly (d-lactic acid) (PLA), and poly (lactic-co-glycolic acid). Among these formulations, the PLA-based scaffolds demonstrated the highest cumulative TGFB1 release over the study period. In vitro cell studies demonstrated that TGFB1-SGnP-PLA fibrous scaffolds supported the proliferation of BMSCs and enhanced chondrogenic differentiation. Transcript expression analysis of BMSCs seeded on TGFB1-SGnP-PLA fibrous scaffolds induced for chondrogenesis revealed an upregulation of chondrocyte-associated markers, including *SOX9*,* ACAN*,* COL2A1*, and *COL1A1*.

**Conclusion:**

This study demonstrates the potential of using SGnPs to protect and deliver chondrogenic induction molecules from electrospun fibrous scaffolds in a sustained manner, promoting the chondrogenic differentiation of BMSCs in cartilage tissue engineering.

Lay Summary.

Researchers have developed advanced biomaterial scaffolds that release bioactive molecules to enhance tissue regeneration. These “smart scaffolds” provide a three-dimensional environment for cell growth and localized cues to support biological functions. Utilizing sugar glass nanoparticles (SGnPs) to encapsulate growth factors like TGFB1, electrospun fibrous scaffolds incorporating TGFB1-SGnPs were crafted to assess their effectiveness in supporting the activity of human bone marrow-derived mesenchymal stem/stromal cells (BMSCs). Results showed that TGFB1, particularly from TGFB1-SGnP-PLA scaffolds, significantly promoted BMSC proliferation and chondrogenic differentiation, as evidenced by increased markers associated with cartilage cells. This innovative approach demonstrates considerable potential for advancing cartilage tissue engineering and offers a new therapeutic strategy for conditions such as osteoarthritis, enhancing tissue repair and regeneration.

## Introduction

Biomaterial scaffolds designed for the controlled release of bioactive molecules provide structural support to cells and guide tissue regeneration while delivering therapeutic agents in a regulated manner. Various strategies have been developed to achieve the controlled release of bioactive molecules, including growth factors, cytokines, and small-molecule compounds, by physically entrapping or chemically binding them to the material. For example, one method employs biodegradable materials that degrade over time, enabling the continuous release of encapsulated molecules [1]. Another approach incorporates micro- or nano-particles within the scaffold, which act as reservoirs for the bioactive molecules [2]. The release rate can be controlled by adjusting particle size and composition. Despite these advancements, significant challenges remain, including achieving precise control over release kinetics [3], preserving the bioactivity of incorporated molecules during fabrication and release [4], and ensuring the long-term stability of the scaffold structure [5].

Electrospinning is a versatile technique for producing three-dimensional fibrous scaffolds that closely mimic the structure and morphology of collagen fibrils found in various tissues [6, 7]. Because of this resemblance, electrospun scaffolds are widely used to support cell culture, particularly of stem cells, in tissue engineering applications [8]. Modulating stem cell activity is critical due to their capacities for self-renewal and multilineage differentiation, ensuring a continuous supply of cells for repair and to differentiate into a range of cell types needed for specific tissues. These capabilities help address key challenges in tissue engineering, such as the limited availability of viable cells for regeneration. Among various stem cells, bone marrow-derived mesenchymal stem/stromal cells (BMSCs) are especially valuable and have been broadly applied in regenerative medicine. For example, directing BMSCs to undergo chondrogenic differentiation enables the scalable production of chondrocytes for cartilage repair [9]. By selecting different polymers and precisely controlling electrospinning parameters, electrospun scaffolds can be tailored to support the adhesion, proliferation, and differentiation of BMSCs [10, 11]. Additionally, electrospun scaffolds can be engineered for the controlled delivery of growth factors, effectively enhancing stem cell activities, including differentiation [12]. Studies have demonstrated the efficacy of incorporating specific growth factors, such as the BMSC-affinity peptide E7 and recombinant human transforming growth factor-beta 1 (TGFB1), into coaxial polycaprolactone (PCL) electrospun scaffolds, promoting chondrogenic differentiation in BMSCs [13]. However, the use of organic solvents in conventional electrospinning poses significant challenges, as these solvents can denature or irreversibly damage growth factors, compromising their biological activity [6, 14]. To address this limitation, growth factors are increasingly being incorporated into carrier systems, such as nanoparticles, which protect them from direct solvent exposure during the electrospinning process and ensure their effective delivery [14, 15].

In our previous study by Giri et al. [16], we introduced the fabrication of reverse micelle sugar-glass nanoparticles (SGnPs) as a novel strategy for incorporating and stabilizing bioactive proteins. Building on that foundation, the current study investigates not only protein encapsulation but also the preservation of protein functionality in environments relevant to scaffold fabrication, particularly during exposure to harsh organic solvents. To this end, horseradish peroxidase (HRP) was employed as a model protein due to its well-characterized enzymatic activity, which enables sensitive and quantitative assessment of functional activity following encapsulation. Moreover, HRP has been previously characterized within our SGnP system [16], making it a suitable surrogate for evaluating protein stabilization under solvent exposure.

Preserving protein bioactivity during electrospinning and other solvent-intensive fabrication processes remains a major challenge in the development of biofunctional scaffolds. Organic solvents such as chloroform are known to denature proteins by disrupting their native structure and promoting aggregation. To counteract these effects, our approach leverages trehalose-(D)-anhydrous as a stabilizing agent within the SGnPs. Trehalose is widely used in the pharmaceutical industry for protein stabilization, particularly during freeze-drying [17], and has been shown to be effective in protecting proteins in dehydrating and solvent-rich environments. The physicochemical basis of this protective effect is explained by two complementary mechanisms. First, the water replacement hypothesis suggests that trehalose stabilizes proteins by replacing the hydrogen bonds typically formed between water and the protein surface. Instead, trehalose forms direct hydrogen bonds with the protein, thereby preserving its native conformation in the absence of water [18, 19]. Second, trehalose forms a highly viscous, glassy matrix with a high glass transition temperature. This vitrified state immobilizes the encapsulated protein, reducing molecular mobility and thereby slowing or preventing unfolding, aggregation, or other destabilizing reactions that can compromise biological activity [18, 20]. This study demonstrates that SGnPs, through these two protective mechanisms, facilitate protein incorporation into fibrous scaffolds and preserve protein activity during fabrication processes that typically inactivate sensitive proteins.

The conventional approach to inducing stem cell activity typically relies on the direct administration of growth factors in cell culture medium. However, this method has limitations, as pulse induction often fails to provide the consistent and sustained stimulation necessary for effective cellular responses, unlike continuous induction [21]. This inconsistency can reduce the efficiency of growth factor-induced cell activities, such as proliferation and differentiation. To overcome this challenge, biomimetic “smart scaffolds” have been developed to enable controlled and continuous release of growth factors. These scaffolds mimic the natural role of the extracellular matrix (ECM) by acting as localized reservoirs for growth factors, facilitating their gradual and sustained presentation to cells. This approach enhances the physiological relevance of growth factor delivery and improves induction efficiency compared to traditional methods [17, 22]. In this study, we explored the potential of freeze-dried SGnPs as carriers for key growth factors involved in chondrogenic differentiation. The SGnPs were fabricated, characterized, and subsequently integrated into electrospun fibers composed of various biodegradable polymers. This approach enabled the development of a smart scaffold system designed to mimic the temporally controlled release of growth factors, thereby enhancing the induction of chondrogenic differentiation in BMSCs.

## Materials and Methods

### Synthesis of Protein-Encapsulated SGnPs

**Fig. 1:**
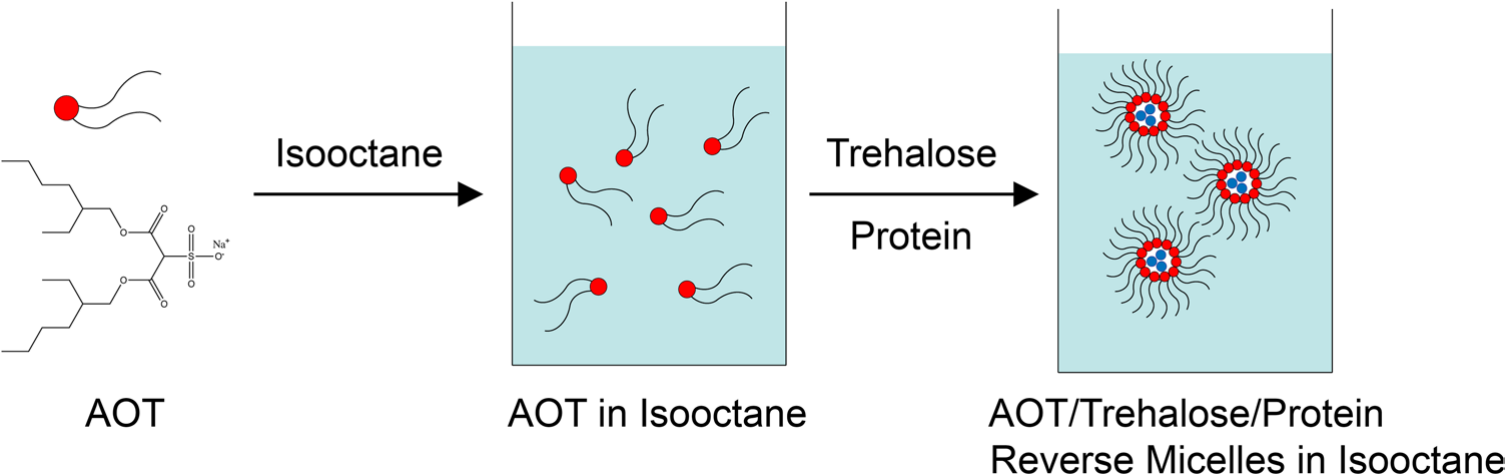
Schematic of reverse micelle SGnPsynthesis. Illustration of the SGnP fabrication process, starting with the formation of AOT/trehalose/protein reverse micelles in isooctane, followed by the freeze-drying of water/AOT/Isooctane emulsions

SGnPs were synthesized using a freeze-drying method that forms sugar-based inverse micelles to encapsulate target proteins, as previously described [16] and shown in Fig. 1. Briefly, 1.6 g of the anionic surfactant sodium dioctyl sulfosuccinate (AOT) was dissolved in 12 mL of isooctane (Sigma Aldrich, MO) to prepare a 0.3 M solution in a 50 mL centrifuge tube. To study the effect of protein loading on SGnP size, bovine serum albumin (BSA) was used as a model protein. A 0.4-mL aqueous solution containing BSA at different amounts (0.133 mg, 0.665 mg, 1.33 mg, or 6.65 mg) was prepared along with excipients to stabilize the protein, including trehalose-(D)-anhydrous (Alfa Aesar, MA), Tween-20 (Sigma Aldrich, MO), CaCl₂ (Sigma Aldrich, MO), and distilled water. The mass ratio of protein to trehalose was maintained at 1:500, following our established protocol [16] to ensure sufficient sugar-glass coating around the protein within the SGnPs. The solution maintained a molar ratio of [H₂O]/[AOT] = 10. The mixture was vortexed for 1 min, then rapidly frozen by dispensing it into a 50 mL centrifuge tube positioned over liquid nitrogen. This flash-freezing step solidified SGnPs with the formation of a stabilizing surfactant and sugar coating around the encapsulated proteins. The frozen nanoparticles were lyophilized overnight. After lyophilization, the nanoparticles were washed with 20 mL of fresh isooctane and centrifuged at 400 g for 10 min. This washing step was repeated four times to effectively remove excess AOT, which is readily soluble in isooctane. The resulting SGnPs were either used immediately or stored at − 80 °C in isooctane under desiccation.

### Characterization of SGnPs

The nanoparticle dispersion in isooctane was analyzed using dynamic light scattering (DLS) with a backscattering angle of 172°, employing a Zetasizer Nano-ZSP instrument (Malvern Instruments, UK). The morphology and size of the nanoparticles were characterized using scanning electron microscopy (SEM) with a Zeiss Auriga Focused Ion Beam FE system (Zeiss, USA). Prior to imaging, SGnPs that had been dispersed in isooctane and subsequently dried were sputter-coated with a 5-nm-thick gold layer. The average diameter of the SGnPs was determined by measuring 100 randomly selected nanoparticles from the SEM images using ImageJ software (National Institutes of Health, Bethesda, MD). The polydispersity index (PDI) of the SGnPs was then calculated by squaring the coefficient of variation of the particle diameters. The formula used was PDI = (*σ*/*μ*)^2^, where *σ* is the standard deviation and *μ* is the mean of the measured particle diameters [23].

### Protein Incorporation and Functional Retention in SGnPs Exposed to Organic Solvents

HRP was used as a model protein to evaluate the effect of the surfactant-to-stabilizing sugar ratio on the amount of HRP loaded into SGnPs. Specifically, AOT to trehalose weight ratios (mg:mg) of 1600:43, 530:43, and 1600:129 in 12 mL isooctane were tested, while maintaining a constant HRP-to-trehalose mass ratio of 1:500. The percentage of HRP encapsulated into SGnPs was determined by measuring the concentration of unencapsulated HRP remaining in the supernatant and the amount loaded into SGnPs, using spectrophotometry at 450 nm with the SIGMAFAST o-phenylenediamine dihydrochloride (OPD) substrate kit (Sigma-Aldrich, St. Louis, MO).

Furthermore, the activity of HRP released from SGnPs after exposure to isooctane and chloroform was assessed to evaluate the ability of SGnPs to protect protein functionality under harsh solvent conditions encountered during fabrication and electrospinning. For this, the enzymatic activity of HRP encapsulated in SGnPs (HRP-SGnPs) and free HRP in buffer (HRP-Buffer) was measured before and after solvent exposure. The extent of functional retention was determined by comparing the residual activity of each group to their respective baseline values. HRP samples were mixed with the organic solvent, vortexed to ensure uniform mixing, and incubated at room temperature for 30 min. Residual enzymatic activity was then quantified using the SIGMAFAST OPD substrate assay (Sigma-Aldrich, St. Louis, MO).

### Fabrication and Characterization of Electrospun Fibrous Scaffolds with Growth Factor-Encapsulated SGnPs

Electrospun scaffolds loaded with growth factor-encapsulated SGnPs were fabricated using three different polymers: poly(D-lactic acid) (PLA, Polysciences, PA), polycaprolactone (PCL, Sigma Aldrich), and poly(D-lactic-co-glycolic acid) 85:15 (PLGA, Alkermes, OH), following a previously established protocol [24]. PLA, PCL, and PLGA 85:15 were dissolved in dichloromethane (DCM, Sigma-Aldrich) to prepare polymer solutions, using concentrations previously optimized in our lab for electrospinning. The corresponding molecular weights, polydispersity indices, and polymer concentrations are summarized in Table 1. To load the scaffolds, 500 μL of TGFB1-encapsulated SGnP dispersion in DCM was added to 3.5 mL of the polymer solution and vortexed for 1 min to mix thoroughly. Based on our previous findings, the ratio of polymer to growth factor protein was kept at 100 to 1. The electrospinning parameters were adjusted for each polymer: the flow rate was maintained at 2.5 mL/h using 18-gauge needles, with tip-to-collector distances of 15 cm (PLA), 18.5 cm (PCL), and 20 cm (PLGA). Applied voltages were 15 kV for PLA and PLGA, and 14 kV for PCL. Scaffolds were collected on aluminum foil and vacuum-dried to remove residual solvent.

**Table 1:** Biodegradable polymer properties and optimal electrospinning concentrations

Polymer type	Molecular weight (g/mol)	Polydispersity index (PDI)	Concentration (% w/v)
PLA	100,000 (Mw)	1.7	13
PCL	80,000 (Mn)	1.6	17
PLGA 85:15	123,000 (Mw)	1.9	20

*Mw* weight average molecular weight, *Mn *number average molecular weight

The morphology of the electrospun fibrous scaffolds was characterized using SEM with a LEO 1530–2 FESEM/EDS instrument (Zeiss, USA). Before imaging, the samples were sputter-coated with a 5 nm thick layer of gold for 1 min to enhance conductivity and improve image quality. The average fiber diameter was determined by measuring 100 randomly selected nanofibers from the SEM images using ImageJ software.

### Release of Growth Factors from SGnP Fibrous Scaffolds

Scaffolds made from PLA, PCL, and PLGA fibers with TGFB1-encapsulated SGnPs were tested to compare their ability to release bioactive TGFB1 over time. The loaded fibrous scaffolds were incubated in 5 mL of PBS at 37 °C to measure the sustained release of growth factors. At each time point, 600 μL of the PBS solution was collected for analysis and replaced with an equal volume of fresh PBS to maintain constant conditions. The concentration of active TGFB1 in the collected samples was quantified using human TGFB1 ELISA kit (RayBiotech, GA), providing a detailed release profile over time. The cumulative release of TGFB1 from the scaffolds was tracked for a total duration of 39 days. The growth factor release data was used as a screening step to determine which polymer composition and growth factor showed the most effective sustained and cumulative release. The selected combination was then used for further assessment with MSCs in culture.

### Isolation and Culture of Human BMSCs

BMSCs were obtained from patients undergoing total hip arthroplasty, following a previously published protocol [25]. The procurement of BMSCs was performed in compliance with guidelines and approval from the Institutional Review Board at the University of Wisconsin-Madison. To isolate BMSCs, harvested bone marrow was mixed with culture medium, and bone debris was removed using an 18-gauge needle and syringe. The mixture was centrifuged at 300 g for 5 min, after which the supernatant was carefully discarded. The resulting cell pellet was resuspended in 25 mL of PBS and layered onto 20 mL of Ficoll-Paque (GE Healthcare, IL). The suspension was centrifuged at 500 g for 30 min, and the mononuclear cell layer was collected. These cells were seeded in a T75 flask and cultured in a growth medium composed of low-glucose DMEM (Gibco, NY), 10% fetal bovine serum (FBS, Atlanta Biologicals, GA), and 1% antibiotics (Atlanta Biologicals, AL). The cells were maintained in a 5% CO₂ humidified atmosphere at 37 °C. Upon reaching 70–80% confluency, the cells were trypsinized using 0.05% trypsin/EDTA (Gibco, NY) and replated at a density of 1000 cells/cm^2^. The culture medium was replaced every 3 days to maintain optimal cell growth.

### Live/dead Assay for Cells on TGFB1-SGnP-PLA Fibrous Scaffolds

The viability of BMSCs cultured on TGFB1-SGnP fibrous scaffolds was evaluated. To prepare the culture environment, 24-well plates were coated with a 0.3% solution of poly(2-hydroxyethyl methacrylate) (poly-HEMA, Sigma Aldrich) in ethanol to prevent cell adhesion to the plate surface. Circular TGFB1-SGnP-PLA fibrous scaffolds, 2 cm in diameter, were cut to size and sterilized by ultraviolet irradiation on both sides for 1 h. Following sterilization, the scaffolds were placed into the poly-HEMA pre-coated 24-well plates and secured with sterilized silicon O-rings. The scaffolds were pre-soaked overnight in BMSC culture medium to prepare them for cell seeding. BMSCs were seeded onto the scaffolds at a density of 50,000 cells/cm^2^ and incubated under standard cell culture conditions of 5% CO₂ at 37 °C. The culture medium was refreshed every 3 days. Cell viability on the scaffolds was assessed using a live/dead staining kit (R&D Systems, MN). Stained cells were visualized with confocal microscopy (Nikon A1RS, Japan), allowing detailed examination of the distribution and condition of live and dead cells within the scaffold structure. To calculate the percentage of dead cells, confocal images were analyzed using pixel-based quantification. Green fluorescence (live cells) and red fluorescence (dead cells) were detected and quantified separately.

### Assessment of Cell Proliferation on TGFB1-SGnP-PLA Fibrous Scaffolds

BMSCs were seeded onto TGFB1-SGnP-PLA and SGnP-PLA fibrous scaffolds at a density of 50,000 cells/cm^2^ to evaluate their proliferation. To prevent cell attachment to the plate surface, 24-well culture plates were pre-coated with a 0.3% solution of poly-HEMA in ethanol prior to use for culturing the cell-seeded fibrous scaffolds. DNA content was extracted from the cell-seeded electrospun fibrous scaffolds on days 1, 3, and 7 of the experiment using the Quant-iT PicoGreen dsDNA assay kit (Invitrogen, OR), following the manufacturer’s instructions. This assay quantifies DNA content, serving as an indicator of cell proliferation and growth within the scaffolds.

### Chondrogenic Differentiation of BMSCs on TGFB1-SGnP-PLA Fibrous Scaffolds

To assess the impact of continuous TGFB1 release from SGnP-incorporated fibers on the chondrogenic differentiation of BMSCs, TGFB1-SGnP-PLA and empty-SGnP-PLA fibrous scaffolds seeded with cells were evaluated for their support of chondrogenesis. For comparison, BMSCs seeded on PLA fibrous scaffolds without incorporated SGnPs served as a positive control and were induced for chondrogenesis using a conventional method of administering exogenous TGFB1 in culture. Cells were seeded onto the PLA fibrous scaffolds of these three groups at a density of 250,000 cells/cm^2^ and initially cultured in a medium comprising low-glucose DMEM, 10% FBS, and 1% antibiotics. The scaffolds were maintained under standard conditions of 5% CO₂ at 37 °C. After 24 h, the culture medium was replaced with a chondrogenic induction medium consisting of high-glucose DMEM, 1% antibiotics, ITSM Premix, 0.9 mM sodium pyruvate, 50 μg/mL L-ascorbic acid-2-phosphate, 40 μg/mL L-proline, and 0.1 μM dexamethasone. For the positive control group, 10 ng/mL TGFB1 was added to the induction medium, while the other two groups were cultured without additional TGFB1 supplementation.

### Total RNA Extraction and Transcript Expression Analysis

At days 1, 14, and 21, total RNA was extracted from the cultured cells using the NucleoSpin RNA II Kit, following the manufacturer’s protocol. The concentration and purity of the extracted RNA were assessed using a Nanodrop 1000 Spectrophotometer (Thermo Scientific). The RNA was then reverse-transcribed into complementary DNA (cDNA) using the High-Capacity cDNA Reverse Transcription Kit (Applied Biosystems) according to the manufacturer’s instructions. Quantitative polymerase chain reaction (qPCR) was conducted to evaluate the expression levels of specific mRNA transcripts. The qPCR analysis was performed using iQ SYBR GREEN Premix (Bio-Rad). Primers specific for chondrogenic markers, including *SOX9*, *ACAN*, *COL2A1*, and *COL1A1,* along with the ubiquitin C (*UBC*) gene as the housekeeping control [26], were utilized. The relative expression levels of the target genes were calculated using the 2^-ΔCt method, normalizing the expression of each gene to the housekeeping gene *UBC*.

### Statistical Analysis

Statistical analysis was performed using GraphPad Prism 9 software (GraphPad, Inc., La Jolla, CA, USA). Quantitative data were presented as the mean ± standard deviation. One-way analysis of variance (ANOVA) was used to evaluate group differences, followed by Tukey’s post hoc test for pairwise comparisons. A *p*-value of less than 0.05 was considered statistically significant.

## Results and Discussion

### Selection of Model Proteins for SGnP Platform Development

To support the development and optimization of the SGnP delivery platform, BSA and HRP were initially used as model proteins before transitioning to TGFB1. Their selection was based on widely accepted practices in assay development and protein delivery research [27, 28]. Both proteins are structurally well-characterized, commercially available at high purity, and known for their stability and ease of handling. BSA is commonly used as a model globular protein for calibration, immobilization, and method development, making it a reliable stand-in for general protein behavior [28, 29]. HRP serves as an enzymatic reporter, facilitating the validation of detection protocols [30]. While neither protein mimics the biological activity of TGFB1, their use allowed for methodological validation of key parameters such as protein immobilization, stability, and release. This approach is a standard step in early-stage troubleshooting. The ability of our SGnP delivery platform to achieve controlled protein release was ultimately confirmed using the target recombinant growth factor, TGFB1. With this rationale, we first evaluated the characteristics and performance of BSA- and HRP-loaded SGnPs before proceeding to experiments with TGFB1.

### BSA-Encapsulated SGnPs Are Nanoparticles with a Relatively Uniform Size Distribution

**Fig. 2:**
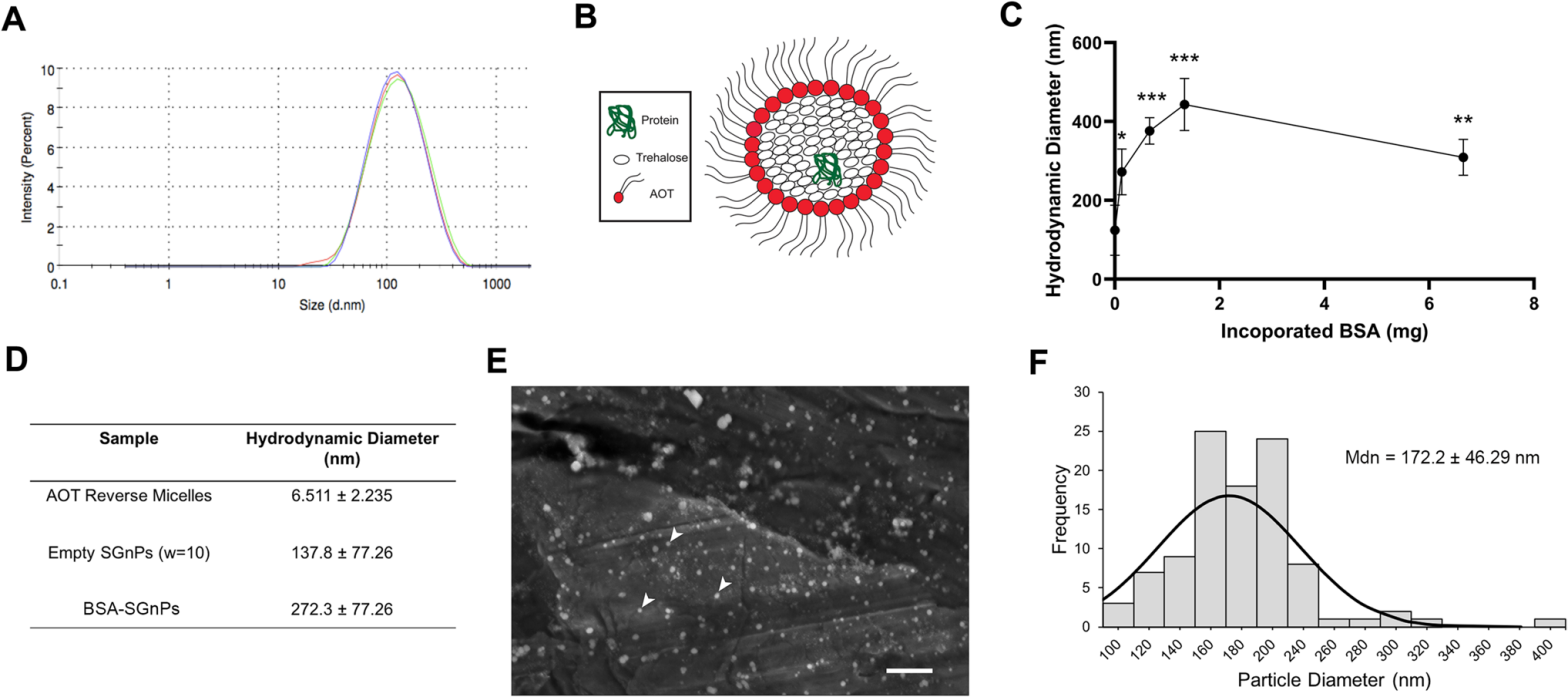
Characterization ofSGnPs. **A** DLS characterization of the average hydrodynamic diameters of SGnPs dispersed in isooctane. **B** Schematic presentation of reverse micelle SGnPs encapsulating target proteins. **C** Impact of incorporated BSA on the hydrodynamic diameter of SGnPs, as measured by DLS. **D** Comparative analysis of average hydrodynamic diameters among AOT reverse micelles, empty SGnPs, and BSA-SGnPs. **E** SEM characterization of dried SGnPs (scale bar = 2 μm). **F** SGnP size distribution measured using ImageJ software

DLS analysis was conducted to measure the hydrodynamic diameter of SGnPs, both with and without protein encapsulation. The results indicated a relatively homogeneous size distribution for unloaded SGnPs, with an average hydrodynamic diameter of approximately 137.8 nm (Fig. 2A). Protein-encapsulated SGnPs were synthesized by flash freezing a trehalose/AOT reverse micellar solution containing the protein of interest, such as BSA (Fig. 2B). To evaluate whether the amount of protein incorporated into SGnPs affects their size, BSA was used as a model protein and incorporated into the nanoparticles in quantities of 0.133 mg, 0.665 mg, 1.33 mg, and 6.65 mg. DLS analysis revealed that the average SGnP diameter increased to 450 nm with 1.33 mg BSA incorporation but decreased to 310 nm with 6.65 mg BSA incorporation (Fig. 2C). These findings suggest a favorable range for protein incorporation between 1.33 mg and 6.65 mg, as higher protein concentrations were associated with a reduction in the average hydrodynamic diameter of SGnPs. Further studies should aim to achieve an optimal balance between maximizing protein loading and maintaining SGnP size that facilitates efficient protein release. The observed size reduction of loaded SGnPs may be attributed to the dual effect of elevated BSA amounts promoting rapid nucleation and enhancing particle stabilization, leading to the formation of a larger number of smaller particles [31]. The influence of albumin concentration on nanoparticle size, where higher concentrations typically yield smaller particles, has been well-documented in protein-based nanoparticle synthesis [32]. Incorporation of 0.133 mg BSA into SGnPs resulted in an average hydrodynamic diameter of 272.3 ± 77.26 nm, compared to an average hydrodynamic diameter of 6.511 ± 2.235 nm for AOT/isooctane reverse micelles and 137.8 ± 77.26 nm for unloaded SGnPs (Fig. 2D). To further validate the DLS measurements, BSA-encapsulated SGnPs in isooctane were dried and analyzed using SEM (Fig. 2E). Measurements of 100 randomly selected BSA-encapsulated SGnPs from the SEM micrographs revealed a relatively homogeneous distribution of nanoparticles, with a few exceeding 300 nm in size (Fig. 2F), likely due to temperature fluctuations during the freeze-drying process. An average size of 172.2 ± 46.29 nm was calculated, which was consistent with, though slightly smaller than, the sizes measured by DLS. The PDI of the BSA-encapsulated SGnPs was calculated using the given formula. With an average diameter of 172.2 nm and a standard deviation of 46.29 nm, the resulting PDI was 0.072. This low value indicates a fairly narrow size distribution, suggesting the nanoparticles are mostly monodisperse [23]. A small number of larger particles may be present, likely due to temperature changes during freeze-drying. These results align with known standards for nanoparticle uniformity, which is important for consistent biodistribution and targeting efficiency [33].

### Optimized SGnPs Enhance Protein Retention and Protection in Solvent Conditions

**Fig. 3:**
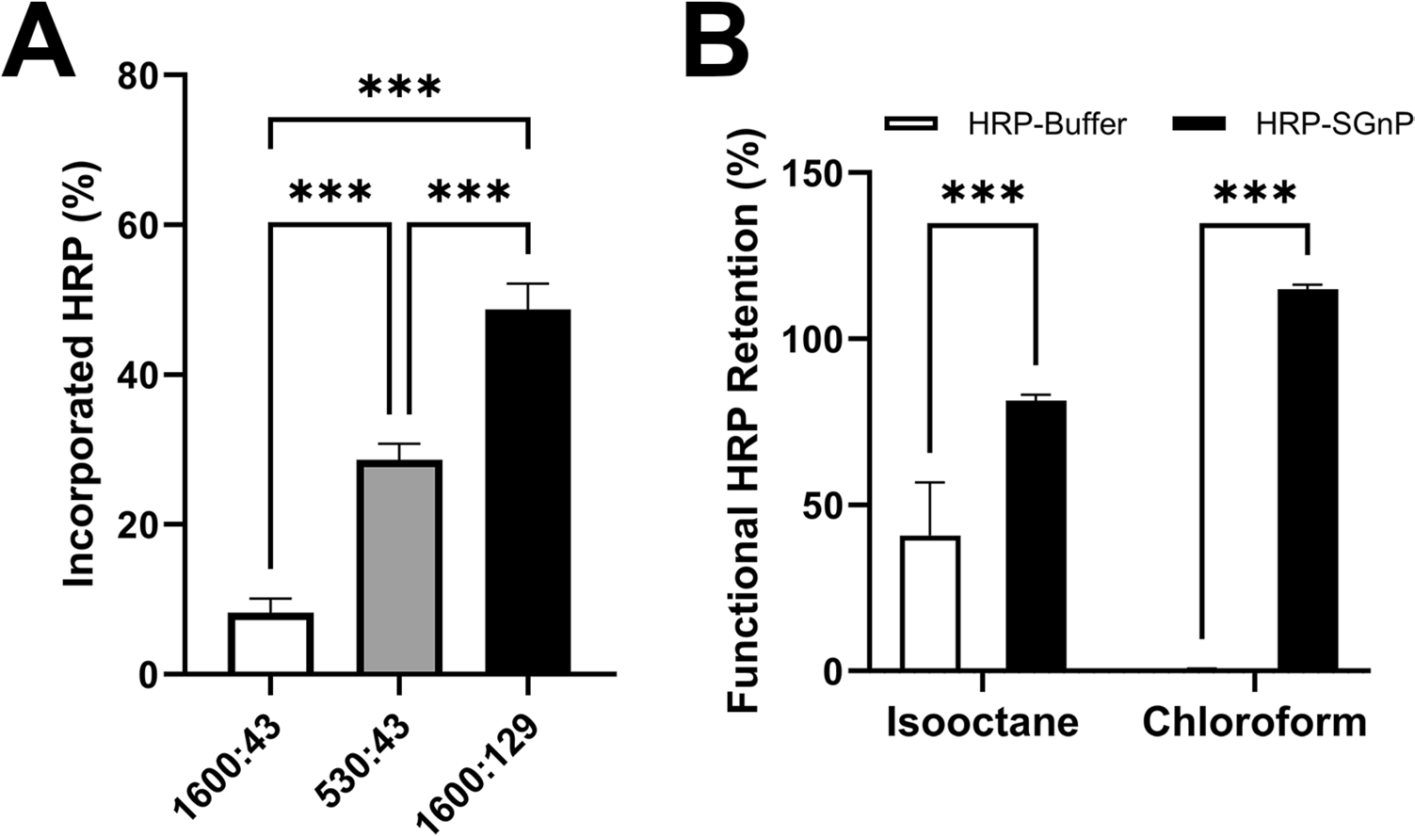
Efficacy of HRP incorporation and protection inSGnPs. **A** Percentages of HRP incorporation in SGnPs produced with varying AOT to trehalose weight ratios (mg:mg), where AOT serves as the surfactant and trehalose as the stabilizing sugar. **B** Activity retention of HRP, with or without SGnP protection, following exposure to organic solvents. *N* = 3; ***p < 0.005

The detection of HRP activity revealed that using 1.6 g of AOT surfactant with 129 mg of trehalose resulted in better retention of functional HRP activity compared to combinations using 1.6 g of AOT with 43 mg of trehalose or 0.53 g of AOT with 43 mg of trehalose. This finding indicates that higher concentrations of AOT and trehalose more effectively protect the protein, presenting an opportunity to further optimize the SGnP synthesis system for improved protein retention (Fig. 3A). Supported by prior research, one possible explanation for the stabilization of proteins with increased trehalose concentration is that trehalose forms a glassy matrix around the proteins, thereby protecting them from solvents [34, 35]. Another widely accepted mechanism is the water replacement hypothesis, in which trehalose forms hydrogen bonds directly with the protein, replacing weaker protein-water interactions with stronger protein–sugar bonds to stabilize the protein’s functional conformation and preserve its activity [18, 19]. Additionally, HRP-SGnPs showed significantly higher enzyme activity in isooctane and chloroform than HRP-Buffer (Fig. 3B), suggesting that SGnPs effectively protect the functional activity of proteins in various harsh solvent environments.

### SGnP Fibrous Scaffolds Exhibit a Uniform Fiber Distribution

**Fig. 4:**
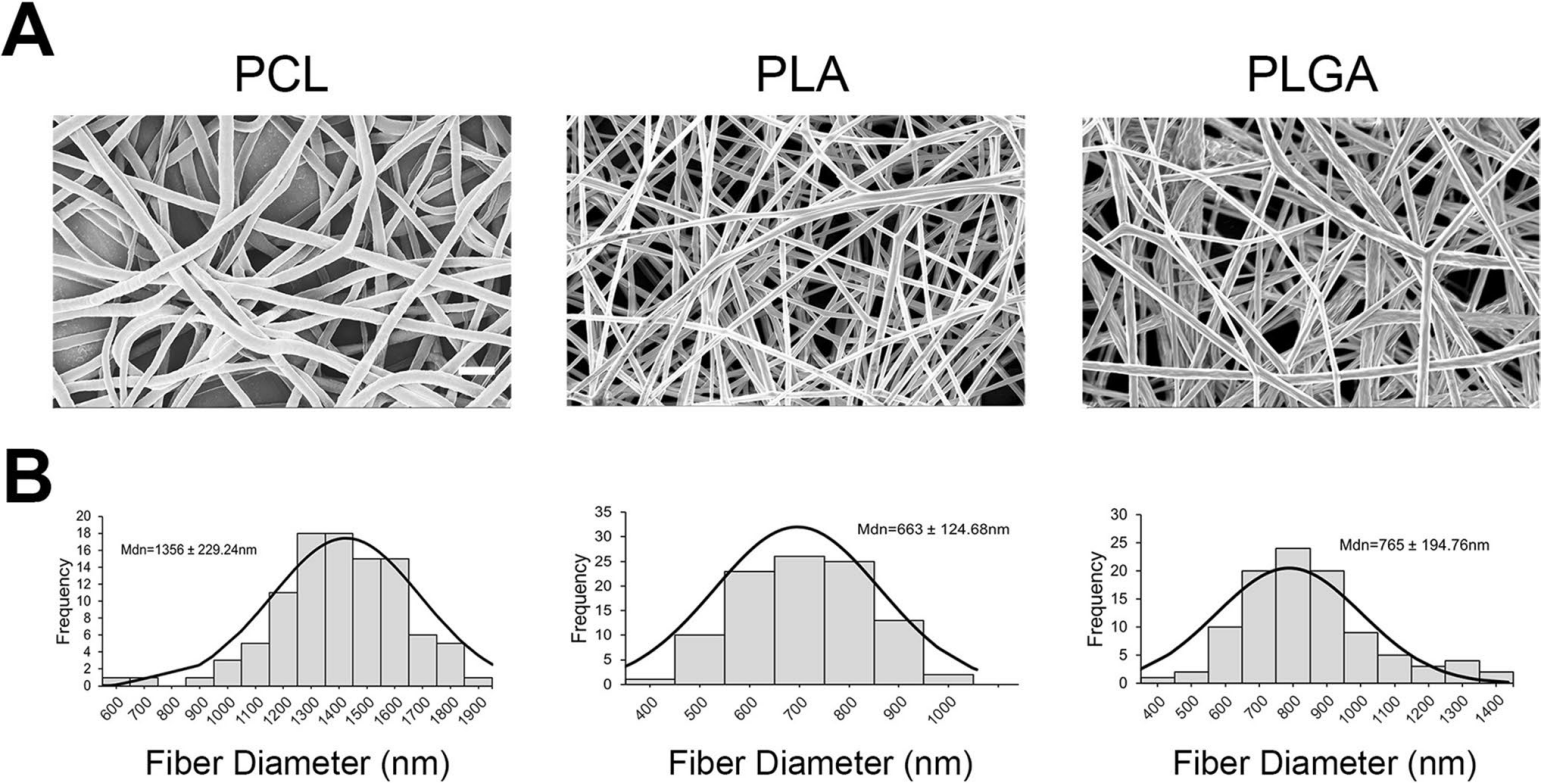
Morphology and fiber size distribution of SGnPfibrous scaffolds.** A** Morphological characterization of electrospun PLGA, PLA, and PCL fibers incorporating TGFB1-SGnPs, analyzed by SEM microscopy (scale bar = 4 μm). **B** Median fiber diameters and their distribution determined by measuring 100 randomly selected individual fibers using ImageJ software

SGnPs were incorporated into polymeric solutions and subsequently electrospun to generate SGnP fibrous scaffolds. SEM analysis of PLGA, PLA, and PCL SGnP fibrous scaffolds revealed a predominantly uniform distribution of fibers without visible beads (Fig. 4A). The median fiber diameter of SGnP-incorporating PCL, PLA, and PLGA fibrous scaffolds was measured to be 1356 ± 229.24 nm, 663 ± 124.68 nm, and 765 ± 194.76 nm, respectively, using ImageJ software. The distribution of fiber diameters followed an approximately normal distribution (Fig. 4B), indicating that the electrospinning parameters for SGnP fibrous scaffolds were optimized specifically for a DCM solvent system. Previous findings have shown that the boiling point and polarity index of DCM contribute to its suitability for this application. First, DCM has a significantly lower boiling point (40 °C) compared to other commonly used solvents such as dimethylformamide (DMF; 153 °C). DCM’s lower boiling point enables rapid solvent evaporation during electrospinning, which is critical for promoting solidification of uniform polymer fibers and minimizing residual solvent content in final electrospun fibers [36]. Second, DCM’s relatively low polarity index (PI) plays a crucial role in preserving protein functionality during solvent exposure in scaffold fabrication. The PI reflects a solvent’s ability to dissolve or interact with polar molecules such as trehalose. High-PI solvents can dissolve trehalose, potentially interfering with its protective role and compromising its stabilizing effect on encapsulated proteins. In contrast, low-PI solvents are less likely to interfere with trehalose, thereby better preserving the protective microenvironment around the proteins [16]. For reference, DMF has a higher PI of 6.4, chloroform has a PI of 4.1, and DCM has a lower PI of 3.1 [37, 38]. Taken together, DCM’s low boiling point and low polarity make it an optimal solvent for fabricating SGnP-based electrospun scaffolds, promoting both uniform fiber morphology and high retention of protein bioactivity.

### SGnP Fibrous Scaffolds Sustainably Release TGB1

**Fig. 5:**
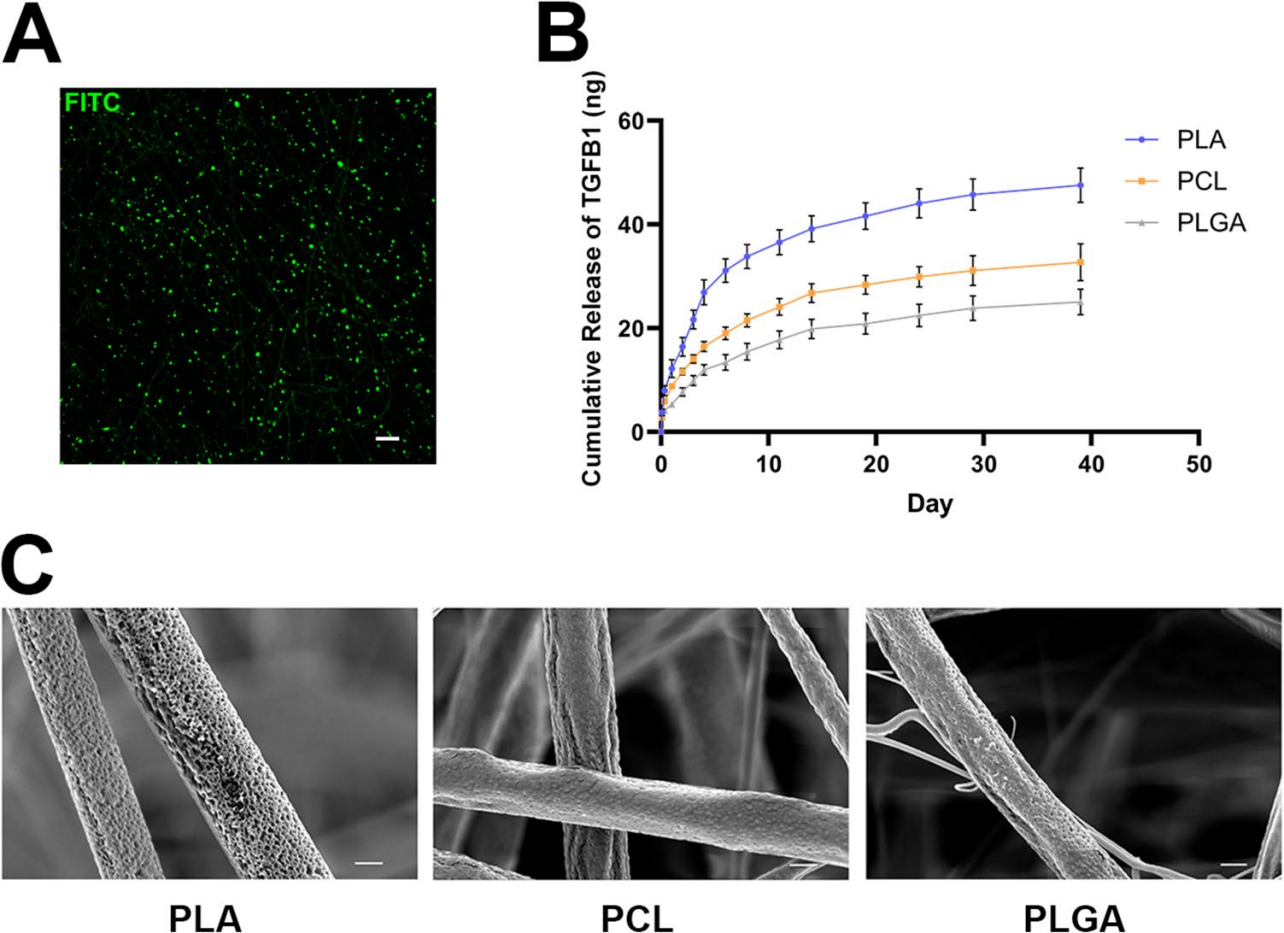
Release of growth factors from SGnP fibrous scaffolds. **A** Electrospun PLA fibers incorporating FITC-SGnPs imaged by laser confocal microscopy (Scale bar = 20 μm). **B** Cumulative release of TGFB1 from SGnP-PLA, SGnP-PCL, and SGnP-PLGA fibrous scaffolds, quantified by ELISA. **C** SEM images showing the morphology of degraded SGnP-PLA, SGnP-PCL, and SGnP-PLGA fibers after 24 h in PBS (scale bar = 0.5 μm)

To visualize SGnPs within fibrous scaffolds, FITC was encapsulated into the SGnPs. Polymer solutions containing these fluorescently labeled SGnPs were electrospun into fibers. Confocal microscopy confirmed the presence of FITC-SGnPs embedded within the electrospun fibers (Fig. 5A). Notably, a portion of the FITC-SGnPs was localized on the surface of the fibers rather than being fully embedded within the fiber matrix. Given the chondrogenic induction potential of the growth factor TGFB1 [25], we characterized its release kinetics from SGnPs embedded within fibrous scaffolds undergoing polymer biodegradation, in preparation for subsequent biological assessment. We fabricated PLA, PCL, and PLGA electrospun fibers incorporating TGFB1-SGnPs and incubated them in PBS solution over a 39-day period. Cumulative TGFB1 release, measured by ELISA, revealed sustained release profiles across all three scaffold types, with PLA scaffolds exhibiting the highest release, followed by PCL and then PLGA (Fig. 5B). TGFB1 release from PLA fibers was approximately 1.9-fold greater than from PLGA fibers and 1.45-fold greater than from PCL fibers over the 39-day period. Specifically, the cumulative release from PLA reached 47.45 ng per scaffold, compared to 32.74 ng for PCL and 25.06 ng for PLGA. Biologically, this difference is significant, as prior studies have demonstrated that TGFB1 concentrations in the range of 1–10 ng/mL are sufficient to induce chondrogenic differentiation in BMSCs [39]. The cumulative TGFB1 levels released from PLA fibers in our system exceed this threshold, thereby fulfilling the design criterion for SGnP fibrous scaffolds to provide sustained TGFB1 delivery necessary for chondrogenic induction. SEM imaging after 24 h of incubation showed distinct early-stage morphological changes for each polymer type (Fig. 5C). PLA fibers developed surface pores and slight swelling, features commonly associated with hydrolytic surface erosion in PLA-based materials [40]. In contrast, PCL fibers developed rough, wrinkled areas on the surface, which reflects PCL’s well-documented slow hydrolytic degradation [41]. PLGA fibers exhibited surface pitting and shallow cracks, reflecting the onset of bulk erosion characteristic of PLGA degradation [42]. These observations match the known patterns of how each polymer’s structure changes during early hydrolytic degradation, as confirmed in our prior study [24].

We observed that TGFB1 release from PLA fibers was higher than from PCL fibers, and both were higher than from PLGA fibers. Notably, this release order does not align with well-established polymer degradation rates reported by several groups [43–45], including our own [24], in which PLGA degrades the fastest, followed by PLA, and then PCL. This apparent discrepancy highlights an important point: the release kinetics of encapsulated proteins in electrospun fibers do not always correlate directly with the bulk degradation rates of the polymers. Instead, release is governed by multiple, interrelated factors including polymer hydrophobicity [46] and crystallinity [47], fiber morphology [48], protein distribution [49], and diffusion kinetics [50]. In this study, the observed release pattern is predominantly controlled by the degradation mechanism of each polymer and its interaction with the aqueous environment. Both PLA and PCL undergo surface erosion [40]. Compared to PCL, PLA is less hydrophobic and less crystalline, allowing water to penetrate the fibers more readily [44]. This promotes faster hydrolysis and a higher rate of fiber breakdown, resulting in more rapid TGFB1 release. In contrast, the greater hydrophobicity and crystallinity of PCL make it more resistant to water penetration and slow its degradation, leading to a lower TGFB1 release rate. PLGA degrades mainly via bulk erosion [51], where water infiltrates the fiber interior and degradation proceeds throughout the material. Although PLGA is known to be more hydrophilic and amorphous, characteristics that typically correlate with faster degradation, our data showed that PLGA fibers released TGFB1 at the slowest rate. This observation is consistent with a prior study by Chou and Woodrow, which demonstrates slower drug release from PLGA fibers compared to PCL [52]. An additional report has shown that incorporating PLGA into PLA-based scaffolds reduces the overall rate of drug release [53].

In addition to these intrinsic material properties, degradation-induced morphological changes may also contribute to the slower release profile observed with PLGA. Our previous work has shown that PLGA electrospun fibers undergo significant swelling when incubated at 37 °C in an aqueous environment, which can cause pore collapse and a reduction in overall structure porosity [24]. This structural change likely restricted protein diffusion and contributed to the lower TGFB1 release observed in PLGA scaffolds. While the degradation behavior of a polymer is a fundamental factor, it alone does not dictate protein release kinetics. Additional factors, such as fiber morphology and structural changes during degradation, must also be considered for the rational design of polymer-based delivery systems. Our previous study demonstrated that parameters such as pH, buffer type, and salt concentration must be carefully optimized for each specific protein during SGnP fabrication to ensure effective protection of the encapsulated payload [16]. Furthermore, the release profiles of proteins can be tailored by modulating the degradation dynamics of the fibrous scaffolds through adjustments in polymer composition. Together, our findings demonstrate the potential of SGnP-based fibrous scaffolds as a versatile platform for sustained and tunable delivery of bioactive proteins, such as the chondrogenic factor TGFB1, through the integration of optimized formulation conditions and scaffold design strategies.

### SGnP Fibrous Scaffolds Support Proliferation and Chondrogenic Differentiation of Human BMSCs

**Fig. 6:**
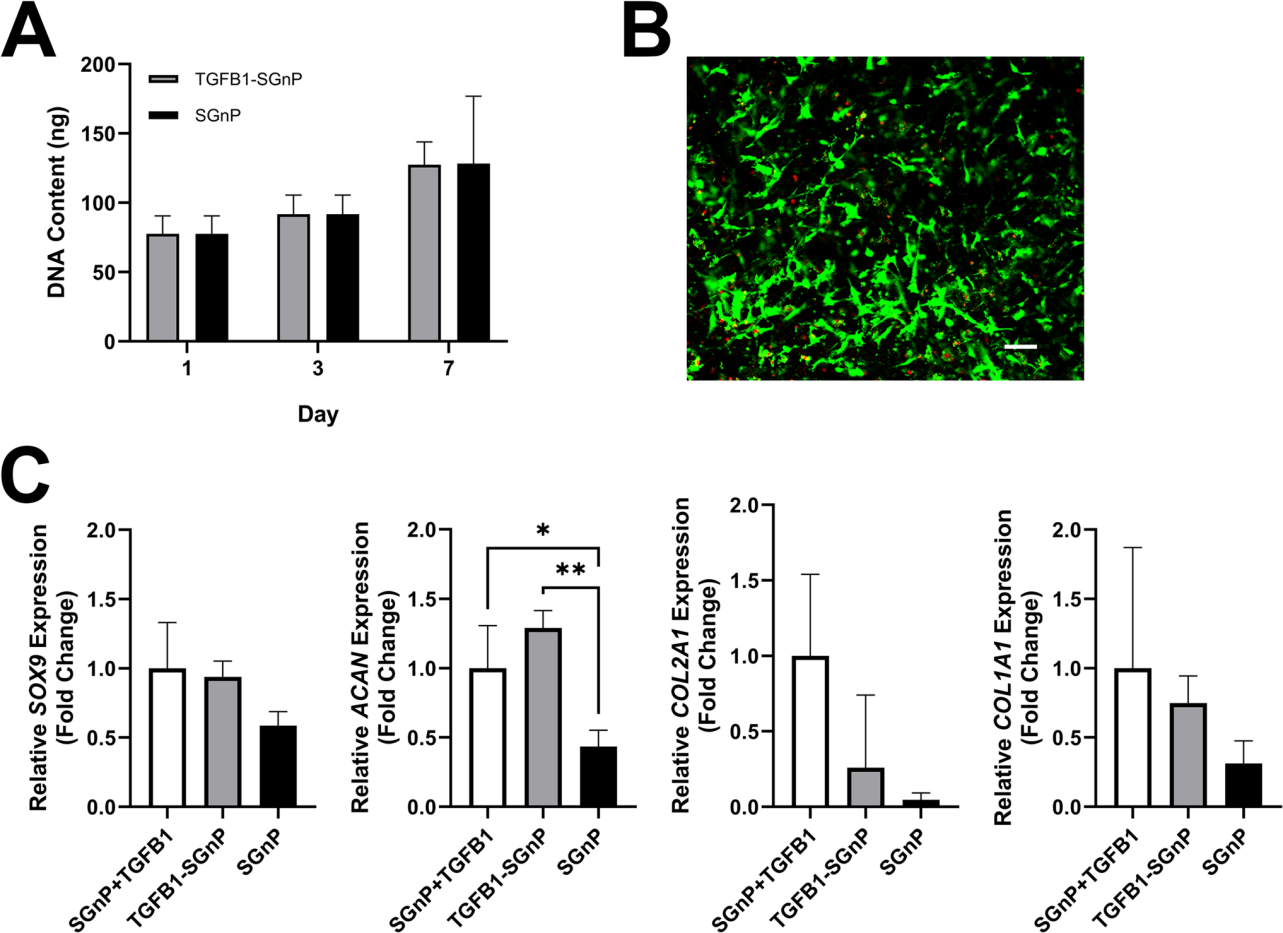
Proliferation and differentiation of BMSCs on TGFB1-SGnP PLA fibrous scaffolds. **A** Proliferation of human BMSCs seeded on TGFB1-SGnP and empty-SGnP fibrous scaffolds over 7 days, quantified by DNA content. **B** Viability of BMSCs on TGFB1-SGnP fibrous scaffolds, assessed by live/dead assay and imaged with confocal microscopy (scale bar = 25 μm). **C** Relative expression levels of cartilage-associated markers in BMSCs seeded on TGFB1-SGnP and empty-SGnP fibrous scaffolds, with or without exogenous TGFB1, after 21-day chondrogenic induction. *N* = 3; *p < 0.05, **p < 0.01

We initially explored the capacity of SGnP fibrous scaffolds to support the viability and proliferation of human BMSCs by seeding cells on both TGFB1-SGnP and empty-SGnP PLA fibrous scaffolds (used as the control). DNA content was quantified at 1, 3, and 7 days post-seeding, with day 1 serving as the baseline control for proliferation. The results showed that both scaffold types supported BMSC proliferation, with no significant differences observed between those with and without encapsulated TGFB1 (Fig. 6A). For the live/dead assay, we specifically evaluated the TGFB1-SGnP scaffolds to assess any potential cytotoxic effects of the surfactant or organic solvent residues. After 5 days of culture, approximately 9.65% of the cells were identified as dead based on fluorescence staining in the representative image (Fig. 6B). This percentage was estimated by analyzing the pixel count per red- or green-stained cell within this image, yielding approximately 149 dead cells and 1395 live cells. While Fig. 6B represents a single image, similar viability trends were observed across replicate samples. These findings suggest that the scaffold components did not exert a detrimental effect on cell viability.

To assess their capability for supporting chondrogenic induction, we seeded human BMSCs on TGFB1-SGnP fibrous scaffolds (TGFB1-SGnP) alongside two control groups: empty-SGnP scaffolds without TGFB1 (SGnP) as a negative control, and empty-SGnP scaffolds with TGFB1 added directly to the culture medium (SGnP + TGFB1) as a positive control. Quantitative RT-PCR analysis conducted after 21 days showed elevated *SOX9* expression in both the SGnP + TGFB1 and TGFB1-SGnP groups compared to the SGnP group (Fig. 6C). While *ACAN* expression was higher in the TGFB1-SGnP group, it was not statistically significant compared to the positive control, but both groups showed significantly higher *ACAN* levels than the negative control (Fig. 6C). The expression patterns for cartilage-associated collagen markers demonstrated that the SGnP + TGFB1 group had the highest average levels of *COL2A1* and *COL1A1*, followed by the TGFB1-SGnP group and the SGnP group (Fig. 6C). These findings can be further interpreted by considering the biological roles of the main chondrocyte-associated markers assessed in this study. SOX9, ACAN, COL2A1, and COL1A1 each represent a distinct stage or aspect of BMSC chondrogenesis. SOX9 is a master transcription factor that initiates the chondrogenic differentiation of BMSCs and is critical for the activation of ACAN and COL2A1, which encode key matrix components of hyaline cartilage [54–56]. SOX9 activates their expression by binding to regulatory elements within their promoters or enhancers. In contrast, COL1A1, a fibrocartilage marker, is also known to be upregulated during BMSC chondrogenesis [57].

In this experiment, consistent with other previous studies using TGFB1 delivery systems such as alginate microspheres [58], PLGA microspheres [59, 60], and chitosan sulfate microspheres [61], we observed upregulation of key chondrocyte-associated markers, including ACAN and COL2A1, in the TGFB1-SGnP group compared to the SGnP control. The expression trends of these markers in our system aligned with those reported for other delivery platforms, suggesting that the SGnP system induces a comparable chondrogenic response in BMSCs. Notably, ACAN expression was significantly higher than the control after 21 days, indicating enhanced production of cartilage-specific matrix components. These outcomes are important because sustained TGFB1 release, as seen with the SGnP system, promotes BMSC chondrogenesis without the need for repeated exogenous supplementation. Collectively, our findings indicate that the SGnP system can effectively induce BMSC chondrogenesis in culture through the controlled release of TGFB1 from fibrous scaffolds. The upregulation of chondrocyte-associated markers in our system follows a trend similar to that reported for other established TGFB1 delivery systems, further supporting the potential of our approach for scaffold-based cartilage regeneration. While our current results demonstrate effective induction of chondrocyte-associated markers, we are continuing to refine the system. To further enhance COL2A1 expression and optimize the regenerative potential of the SGnP platform, our ongoing work aims to increase TGFB1 release from the scaffolds by encapsulating a greater amount of this growth factor.

## Conclusion

In this study, we demonstrated the fabrication of SGnPs by freeze-drying reverse micellar solutions containing trehalose and proteins, which were then incorporated into polymeric solutions and electrospun into fibers. We used various polymers and solvent systems to produce SGnP fibrous scaffolds with distinct fiber morphologies and degradation kinetics. Our findings confirmed that we successfully fabricated SGnP fibrous scaffolds capable of preserving the functional integrity of proteins such as HRP and TGFB1, despite the presence of organic solvents. Additionally, these scaffolds facilitated the sustained release of chondrogenic growth factor TGFB1 over several weeks. The scaffolds also supported BMSC adhesion and proliferation without the need for additional surface treatments. Importantly, the TGFB1 released from SGnP-incorporated fibers effectively stimulated a cellular response, promoting the chondrogenic differentiation of seeded human BMSCs. This was evidenced by the upregulated expression of *SOX9*, *ACAN*, *COL2A1*, and *COL1A1* in BMSCs cultured on TGFB1-SGnP fibrous scaffolds, highlighting the scaffolds’ capability for localized growth factor delivery to induce cellular responses. In conclusion, SGnP fibrous scaffolds, specifically designed to protect and deliver proteins of interest, have demonstrated the ability to enhance targeted cellular activities. This platform not only preserves the bioactivity of encapsulated proteins but also ensures their controlled and sustained release, mimicking the natural functionality of the ECM in the body. By integrating protein protection, delivery, and bioactivity maintenance into a unified platform, SGnP fibrous scaffolds establish themselves as “smart scaffolds.” Their ability to support the delivery of a diverse range of therapeutic proteins holds great promise for improving outcomes in tissue repair and regeneration.

## Data Availability

All relevant data generated or analyzed in this study can be obtained from the corresponding author on reasonable request.
